# Autoimmune pulmonary alveolar proteinosis developed during immunosuppressive treatment in polymyositis with interstitial lung disease: a case report

**DOI:** 10.1186/s12890-020-1110-5

**Published:** 2020-04-06

**Authors:** S. Sato, K. Akasaka, H. Ohta, Y. Tsukahara, G. Kida, E. Tsumiyama, K. Kusano, T. Oba, T. Nishizawa, R. Kawabe, H. Yamakawa, M. Amano, H. Matsushima, T. Takada

**Affiliations:** 1 Department of Respiratory Medicine, Saitama Red Cross Hospital, 1-5, Shintoshin, Chuo-ku, Saitama, 330-8553 Japan; 2 Uonuma Institute of Community Medicine, Niigata University Medical and Dental Hospital, Minami-Uonuma, Japan

**Keywords:** Pulmonary alveolar proteinosis, Polymyositis, GM-CSF, Immunosuppressive treatment

## Abstract

**Background:**

Pulmonary alveolar proteinosis (PAP) is characterized by the accumulation of surfactant proteins within the alveolar spaces. Autoimmune PAP (APAP) caused by elevated levels of GM-CSF autoantibodies (GM-Ab) is very rarely associated with systemic autoimmune disease. Here we report a case of APAP manifested during immunosuppressive treatment for polymyositis with interstitial lung disease.

**Case presentation:**

A 52-year-old woman treated at our hospital because of polymyositis with interstitial pneumonia had maintained remission by immunosuppressive treatment for 15 years. She had progressive dyspnea subsequently over several months with her chest CT showing ground-glass opacities (GGO) in bilateral geographic distribution. Her bronchoalveolar lavage fluid with cloudy appearance revealed medium-sized foamy macrophages and PAS-positive amorphous eosinophilic materials by cytological examination. We diagnosed her as APAP due to an increased serum GM-CSF autoantibody level. Attenuating immunosuppression failed to lead GGO improvement, but whole lung lavage (WLL) was effective in her condition.

**Conclusions:**

PAP should be considered as one of the differential diseases when the newly interstitial shadow was observed during immunosuppressive treatment. WLL should be regarded as the treatment option for APAP concurred in connective tissue disease (CTD).

## Background

Pulmonary alveolar proteinosis (PAP) is a rare **syndrome** resulting from the accumulation of lipoproteinaceous materials in the alveoli and terminal airways due to impairment of surfactant clearance by alveolar macrophage [1]. PAP can be classified into several types based on the pathogenesis [2]. Primary PAP is characterized by the disruption of granulocyte-macrophage colony-stimulating factor (GM-CSF) signalling pathway: autoimmune (caused by elevated levels of GM-CSF autoantibodies) or hereditary (due to mutations in encoding GM-CSF receptor subunits). Secondary PAP results from various underlying diseases. Congenital PAP is caused by mutations in genes involved in surfactant production. Interestingly, autoimmune PAP (APAP) is very rarely associated with systemic autoimmune disease. Only a few cases can be found in registry studies and case reports [3–9].. Furthermore, APAP complicated with dermatomyositis/polymyositis was reported only in one case of aminoacyl-tRNA synthetases (ARS) related dermatomyositis [7]. Here we report a case of APAP manifested during long-term immunosuppressive treatment for polymyositis with interstitial lung disease.

## Case presentation

A 52-year-old Japanese woman visited our hospital in 2004 with complaints of dyspnea, dry cough, and myalgia. She had signs of myalgia and muscle weakness of the upper limbs, increased serum creatine kinase (1315 IU/L), and fibrillation potential and short duration neuromuscular unit (NMU) in electromyogram. We diagnosed her as polymyositis based on the diagnostic criteria of the Ministry of Health, Labour and Welfare’s Autoimmune Disease Research Group of Japan. No skin symptoms were observed. Chest CT findings revealed reticular shadows and ground-glass opacities (GGOs) in bilateral lower lobes peripherally from around the bronchovascular bundle. The bronchoalveolar lavage fluid (BALF) with transparent appearance contained 2.4 × 10^5^ cells/mL, 44.6% macrophages, 52.2% lymphocytes, 1.8% neutrophils, and 1.4% eosinophils. Lymphocytes increment was observed but no findings suggesting PAP on cytology. **Microbiological tests for bacteria, fungi, or acid-fast bacilli in BALF were also negative. The titer of GM-Ab was not measured.**

**Fig. 1:**
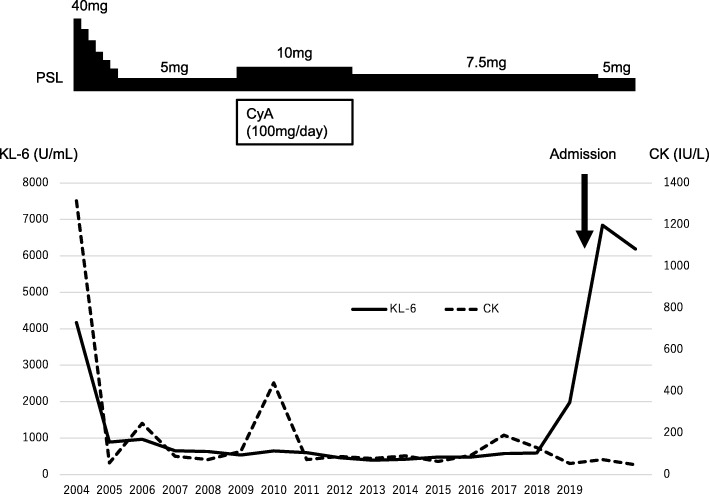
Clinical course. The patient had no recurrence for fifteen years with immunosuppressive treatment after diagnosis with polymyositis and myositis-associated interstitial pneumonia. In 2019 she was admitted to our hospital complaining progressive dyspnea with increased KL-6.

**Fig. 2:**
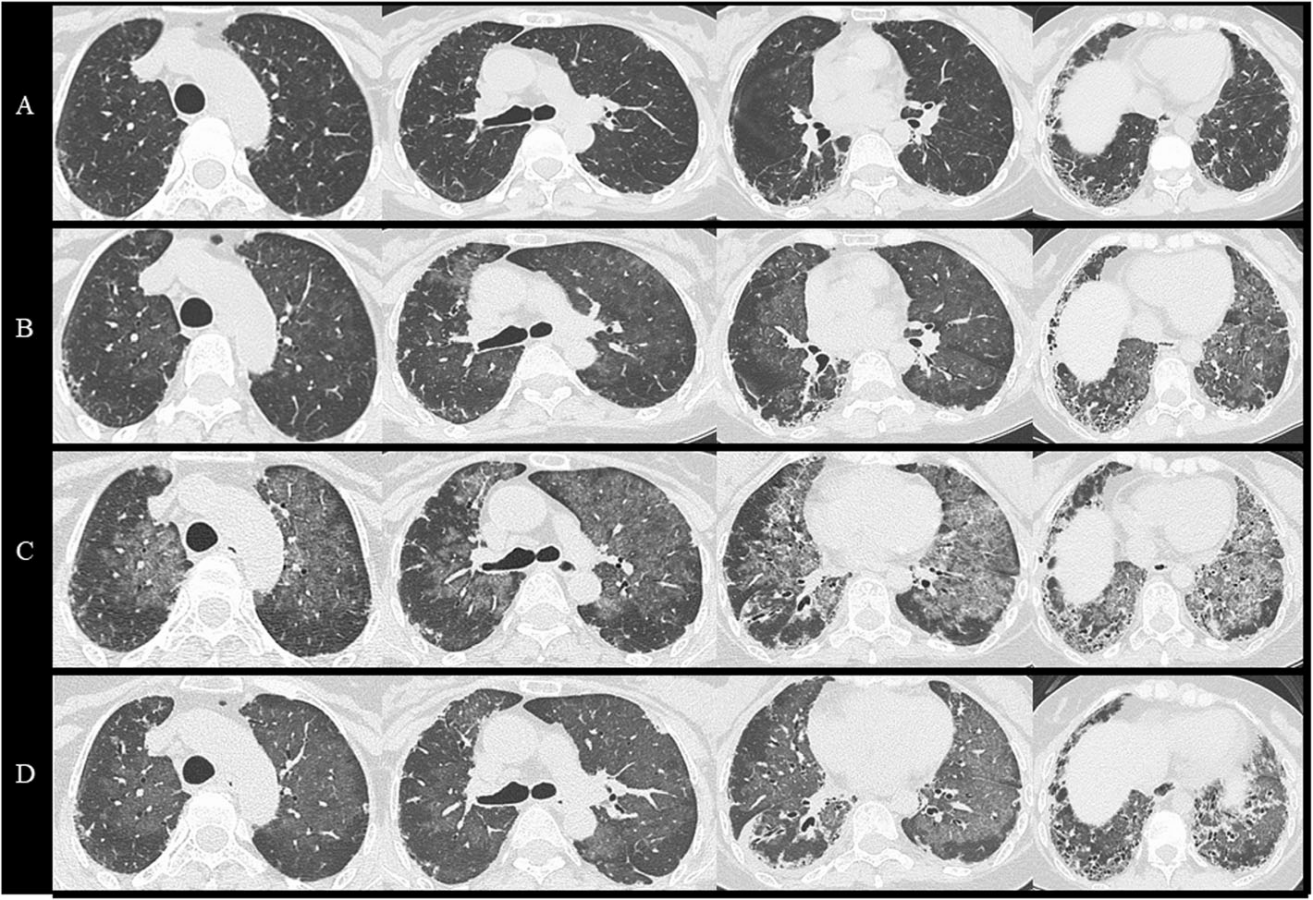
Chest CT findings. **A** Four months before admission, her chest CT showed a subpleural and basal predominant reticular pattern with peripheral traction bronchiectasis. **B** On admission, extensive GGOs with geographic distribution appeared bilaterally. **C** Immediately before WLL, GGOs worsened further in both lung fields four months after the diagnosis. **D** After WLL, GGOs improved significantly with no change in the underlying interstitial pneumonia.

She was diagnosed as polymyositis with interstitial pneumonia. Corticosteroid therapy (**prednisolone**;40 mg/day) immediately improved both myalgia and dyspnea. Serum creatine kinase and KL-6 levels also decreased to normal ranges. As for CT findings, the reticular opacities and GGOs diminished. We gradually tapered **prednisolone** doses and her medical condition had been well-controlled by immunosuppressive treatment over 15 years since the diagnosis (Figs. 1, 2A).

In early 2019, she visited our hospital because of progressive dyspnea and slight productive cough for 4 months under treatment with **prednisolone** 7.5 mg/day (Fig. 1). Chest auscultation revealed fine crackles on inspiration in the bibasilar area. The physical examination revealed no skin lesions such as sclerosis, Gottron’s sign, heliotrope rash, mechanic’s hands, or Raynaud’s phenomenon, and no myalgia, muscle atrophy, or arthralgia. Chest CT showed subpleural reticular opacities predominantly in the lower lobes accompanying contraction and extensive GGOs in geographic distribution (Fig. 2B).

Laboratory examinations revealed significantly high serum levels of KL-6 (6843 U/mL) and SP-D (362 ng/mL). The serum creatine kinase (72 IU/L), aldolase (5.3 IU/L), and C-reactive protein levels (0.27 mg/dL) were within the normal range. The serum anti-ARS antibody level was positive at 115 index, whereas anti-melanoma differentiation-associated gene 5 (MDA-5) antibody level was negative. RNA immunoprecipitation assay indicated the positivity of anti-SS-A/Ro52 and anti-EJ, one of the anti-ARS antibodies. An arterial blood gas analysis showed decreased partial pressure of oxygen (PaO_2_) (74.5 Torr) with elevated alveolar-arterial oxygen gradient (A-aDO_2_) (22.3 Torr). The respiratory function tests showed severely impaired forced vital capacity (FVC) of 0.6 L (28.5%, %predicted) and forced expiratory volume in 1 s (FEV1) of 0.69 L (35.2%, % predicted) with 100.0% of FEV1/FVC ratio. The diffusing capacity of carbon monoxide (DL_CO_) could not evaluate due to low FVC.

The BALF with cloudy appearance contained 3.7 × 10^5^ cells/mL consisting of 85.2% macrophages, 9.6% lymphocytes, 3.6% neutrophils, and 1.4% eosinophils. **Microbiological tests, including** ***Pneumocystis jirovecii*** **polymerase chain reaction (PCR) in BALF, were negative**. A cytological examination of BALF revealed medium-sized foamy macrophages with acellular periodic acid-Schiff (PAS) positive bodies. Transbronchial lung biopsy specimens showed myxoid-appearing organization in alveolar space and alveolar duct, but no amorphous PAS-positive materials. Because these pathological findings strongly suggested PAP, we measured serum GM-CSF autoantibody (GM-Ab) levels and found positive at 80.1 μg/mL (cut off value < 1.0 μg/mL) [10, 11]. Based on these results, we diagnosed her as APAP in myositis with interstitial pneumonia with severity 2 according to the disease severity score (DSS) [3].

**Fig. 3:**
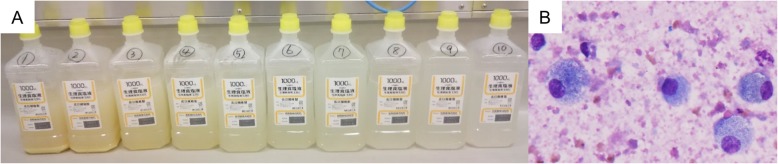
Washed-out recovery fluid of WLL. **A** Gross findings showed a milky appearance with sediment. **B** Microscopic results revealed foamy macrophages backed by eosinophilic proteinaceous granular materials

According to the previous report [7] **prednisolone** dose was decreased from 7.5 mg/day to 5.0 mg/day. However, in a gradual progression of her chest imaging findings (Fig. 2C), we performed whole lung lavage (WLL) 4 months after the diagnosis of APAP. The washed-out recovery fluid revealed milky appearance typical of PAP with sediment. It contained foamy macrophages with a background of eosinophilic proteinaceous granular materials (Fig. 3). After the treatment, the GGOs in both lung fields were remarkably improved on CT (Fig. 2D). We found no obvious recurrence of myositis, interstitial pneumonia, or PAP for the following several months.

## Discussion and conclusions

We experienced a case of APAP complicated with polymyositis accompanying interstitial lung disease. As the interstitial lung abnormality confirmed at first visit day, we considered it to be the myositis-related interstitial pneumonia. **The disorder revealed no cloudy appearance of BALF with no pathological findings suggesting PAP, and resolved with immunosuppressive treatment including corticosteroid. We previously reported that corticosteroid therapy might exacerbate APAP** [12]. **Thus, although the GM-Ab titer was not measured, it is unlikely that the patient had a mild form of APAP when diagnosed as polymyositis with interstitial pneumonia**. The patient developed APAP during the immunosuppressive treatment for polymyositis for 15 years. Attenuation of the immunosuppression did not affect newly appeared GGO, but whole lung lavage (WLL) remarkably improved her CT findings and respiratory condition.

There are very few cases of PAP complicated with connective tissue disease (CTD). Seymour reported only 7 out of 410 PAP cases (1.7%) diagnosed within the period from 1958 to 2002 [1]. According to a report from the Japanese registry, CTD was only found in 3 out of 223 cases of APAP (1.3%, 1999–2006) [3]. We confirmed six cases with CTD complicated with APAP from 1998 to 2019 by searching in the PubMed website (Table 1) [5–9]. All cases were treated by immunosuppressive agents.

**Table 1:** Previously reported cases with autoimmune PAP complicated with CTDs

Case	Author	Age	Sex	Duration	Time to	CTD	CTD	GM-Ab	Regular	PAP
					onset	precedence		(μg/mL)	Treatment	Treatment
1 [5]	Nagasawa	26	F	month to year	2 M	Yes	SLE	10.6	PSL + IVCY	reduce PSL + discontinue IVCY
2 [6]	Yamasue	64	F		1.1Y	Yes	SSc	10.8	PSL + IVCY	reduce PSL + discontinue TAC
3 [7]	Imura	58	F		3.5Y	Yes	DM	1.8	PSL + CyA	reduce PSL
4 [8]	Ito	65	F	year to decade	5Y	Yes	RA	26.1	MTX + SASP	WLL + inhalation GM-CSF
5	Our case	52	F		15Y	Yes	PM	80.1	PSL	reduce PSL + WLL
6 [9]	Sakamoto	65	M		20Y	Yes	UC	62.8	SASP	WLL
7 [8]	Ito	68	F		26Y	Yes	RA	42.3	SASP	WLL + inhalation GM-CSF

*CTD* connective tissue disease, *CyA* cyclosporine A, *DM* dermatomyositis, *GM-Ab* granulocyte-macrophage colony-stimulating factor autoantibody, *GM-CSF* granulocyte-macrophage colony-stimulating factor, *IVCY* intravenous cyclophosphamide, *MTX* methotrexate, *RA* rheumatoid arthritis, *SASP* salazosulfapyridin, *SLE* systemic lupus erythematosus, *SSc* systemic sclerosis, *TAC* tacrolimus, *UC* ulcerative colitis, *WLL* whole lung lavage

Several case reports showed improved CT findings with only corticosteroid reduction [5–7]. We hypothesize that immunosuppressive treatment with corticosteroid might cause alveolar macrophage dysfunction and increase the accumulation of phospholipids in the alveolar air space. Then it is plausible that the use of immunosuppressive agents could promote the development of potential PAP [12–15] and that corticosteroid reduction caused improvement of CT findings.

GM-CSF regulates the clearance of surfactant by alveolar macrophages. GM-Ab, antibodies to GM-CSF neutralize the effect of GM-CSF on alveolar macrophages, resulting in the maturation arrest of those cells and the development of PAP. GM-Ab in APAP only consisted of the IgG isotype [11]. Immunosuppressive drugs with corticosteroids usually decrease the serum level of IgG. Some patients, however, develop APAP under treatment, which implies that IgG of GM-Ab may be produced independently in immunosuppressive therapy.

The 6 cases listed on the Table 1 can be divided into two groups. The first group (Case 1–3) developed APAP in 2 months to 3.5 years during an intensive immunosuppressive treatment and improved by tapering the therapy without WLL. Whereas the other group with our case (Case 4–7) developed APAP in 5 to 26 years during mild immunosuppressive treatment and required WLL. This long-term group showed more elevated GM-Ab levels than the short-term group. GM-Ab was likely produced by continuous GM-CSF stimulation in the long-term remedy for CTD. These results suggest that immunoinflammatory pathology for the development of APAP might be different between these groups.

Physicians rarely consider PAP as a diagnosis when they found unanticipated interstitial opacity during the treatment course of CTDs. Most probable differential diagnoses are as follows: opportunistic infection including *Pneumocystis jirovecii* or CMV pneumonia, a progression of CTD-related interstitial pneumonia, and drug-induced lung injury. **None of these differential disorders was likely in this case because of no positive microbiological tests of BALF, no exacerbation of CTD, and no newly administered drugs**. Kitamura recently reported, however, that APAP occurs more frequently than previous reports [16], which suggests that PAP cases might be overlooked among CTD cases treated with immunosuppressive agents.

**Recently, a high incidence of juvenile idiopathic arthritis-associated lung disease is reported** [17]. **Eighteen patients with the disease showed a pulmonary histopathology pattern of endogenous lipoid pneumonia and PAP without GM-Ab. The heritability of APAP has not been proven and is considered to be unrelated to HLA** [18]. **PAP may be more likely to occur in CTD with or without the GM-Ab. One reason is that we suspect that immunosuppressive drugs could manifest APAP by macrophage dysfunction or opportunistic infection. We may need to be more cautious with CTDs that potentially coexists with PAP** [18].

In the present case, we performed WLL with no significant complications. However, it was also a great concern about postoperative pneumonia, respiratory failure, and acute exacerbation of interstitial pneumonia. In the cases of refractory PAP, rituximab and plasmapheresis were also reported as treatment options [19, 20]. Recently, Tazawa et al. reported that inhaled recombinant human GM-CSF was associated with a modest salutary effect on the laboratory outcome of arterial oxygen tension in a randomized, controlled trial [21]. The efficacy and safety of recombinant human GM-CSF for the PAP with pulmonary fibrosis should be studied.

In conclusion, we experienced a case of APAP associated with polymyositis and myositis-related interstitial pneumonia. This rare concurrence might not a coincidence because of previous reports. Among CTD cases treated with immunosuppressive agents, PAP should be considered as one of the differential diagnosis when unexpected interstitial opacities were observed. WLL is useful as a standard treatment for those cases.

## Data Availability

All the data regarding the findings are available within the manuscript.

## Abbreviations

APAP: Autoimmune pulmonary alveolar proteinosis
ARS: Aminoacyl-tRNA synthetase;
BALF: Bronchoalveolar lavage fluid
CK: Creatine kinase
CTD: Connective tissue disease
CyA: Cyclosporine A
GGO: Ground-glass opacity
GM-Ab: GM-CSF autoantibody
GM-CSF: Granulocyte-macrophage colony-stimulating factor
KL-6: Krebs von den lungen-6
PAP: Pulmonary alveolar proteinosis
WLL: Whole lung lavage
